# Genetic Variants of Echovirus 13, Northern India, 2010

**DOI:** 10.3201/eid1902.111832

**Published:** 2013-02

**Authors:** Harjeet Singh Maan, Rashmi Chowdhary, Akhalesh Kumar Shakya, Tapan N. Dhole

**Affiliations:** Author affiliations: Sanjay Gandhi Postgraduate Institute of Medical Sciences, Lucknow, India (H.S. Maan, A.K. Shakya, T.N. Dhole);; Columbia University, New York, New York, USA (R. Chowdhary)

**Keywords:** Acute flaccid paralysis, nonpolio human enteroviruses, emerging nonpolio HEV, echovirus 13, viruses, India

## Abstract

Nonpolio acute flaccid paralysis is increasing in India. To determine viral causes, we conducted cell culture and molecular analysis identification of nonpolio human enteroviruses associated with acute flaccid paralysis during March–August 2010 in northern India. The predominant nonpolio enterovirus found was echovirus 13, a serotype rarely isolated in India.

Although polio has not been reported from India since January 2011, spurts in the rate of nonpolio acute flaccid paralysis (AFP) are of concern. Therefore, other viral agents associated with AFP need to be identified, especially nonpolio human enteroviruses (HEVs), which are a major cause of neurologic illness.

The classic method for HEV serotypic identification requires virus isolation in susceptible cell cultures, followed by virus neutralization tests. However, the procedure is laborious, slow, and incapable of identifying new serotypes for which no reference antiserum is available (*1*). Nonpolio HEV typing based on viral protein (VP) 1 sequences has been developed and correlates well with antigenically defined serotypes (*2*).

Since 1998, we have been actively involved in polio surveillance and passively reporting nonpolio HEV activity in Uttar Pradesh State, northern India. Most nonpolio HEVs were isolated during the summer–autumn season and identified by using methods based on antigenic serotyping (*3*). We used a method combining a single cell culture passage with VP1 reverse transcription PCR (RT-PCR) and sequencing for rapid identification of nonpolio HEV serotypes. The predominant nonpolio HEV found was echovirus (E) 13, a serotype rarely isolated in India.

## The Study

Nonpolio AFP case-patients showing signs of acute onset of focal weakness or paralysis characterized as flaccid (reduced muscle tone) and preceded by fever were considered for NPEV analysis. A total of 347 fecal specimens collected in Uttar Pradesh during March–August 2010 from children <15 years of age who had AFP symptoms were analyzed by using a modified World Health Organization (WHO) method (4,5).

Fecal specimens were processed according to the WHO protocol (*4*). Human rhabdomyosarcoma and L20B (mouse fibroblast cells expressing the poliovirus receptor) cell lines used for virus isolation were observed for cytopathic effect (CPE). The positive isolates with (enterovirus-like) CPE were tested by using pan-poliovirus and pan-EV RT-PCR (*4*). All poliovirus isolates characterized according to WHO algorithm were excluded from the analysis presented here. Viral RNA was extracted from the first-pass rhabdomyosarcoma cell suspension with the Viral RNA Mini Kit (QIAamp, QIAGEN, Hilden, Germany). Genotyping was done by RT-PCR of a portion of the VP1 gene and sequencing as described (*2*,^6^).

MEGA version 5.05 software (*7*) was used for phylogenetic analysis. Distance matrices of the nucleotide and amino acid sequences were analyzed with the MegAlign program in the Lasergene software package (DNASTAR, Madison, WI, USA).

Of 347 fecal samples analyzed, 73 (21%) cell cultures tested positive by pan-EV 5′ nontranslated region RT-PCR. Nineteen (5%) were pan-poliovirus positive, leaving 54 (16%) nonpolio HEVs for analysis. Genotyping of 45 (83%) EVs by partial VP1 sequencing followed the criteria of Oberste et al. (*2*). CPE was observed in rhabdomyosarcoma cells in the first passage for 33 samples, of which 27 were pan-EV positive, identified as E13 (21 [64%]), E7 (2 [6%]), E4 (1 [3%]), E33 (1 [3%]), and EV75 (2 [6%]); 6 isolates remained untypeable. Of the 21 samples that did not yield CPE in rhabdomyosarcoma cells in the first passage, 18 were pan-EV positive, identified as E25 (4 [19%]), E13 (9 [43%]), coxsackievirus B3 (CVB3; 3 [14%]), CVB6 (2 [10%]). Three isolates could not be identified because of poor quality sequence data.

E13 was the most common nonpolio HEV identified. Because E13 has rarely been isolated in India, we analyzed the Uttar Pradesh E13 isolates for March–August 2010 from 5 adjoining districts of western Uttar Pradesh (Lakhimpur-Kheri, Sitapur, Lucknow, Raebareli, and Hardoi). Clinical findings (Table 1) showed that 20 (67%) of the 30 patients had asymmetric paralysis at onset, including 3 AFP case-patients with fever at onset of paralysis and residual paralysis mimicking poliolike illness; however, when compared with other etiologies, no conclusion was inferred (Table 2).

**Table 1:** Clinical and virologic findings for AFP case-patients from whom echovirus 13 was isolated, Uttar Pradesh, India, 2010*

Clade,† isolate name‡	Month§	District¶	Patient age, y/sex	Disease onset		Paralysis status after 60 d follow-up (residual paralysis)
				Fever	Asymmetric paralysis	
A						
Har01/IND/UP/Echo13/2010	Mar	LNO	3.10/M	–	–	+
Har05/IND/UP/Echo13/2010	Mar	STP	2.9/F	–	–	+
IND-29-2010	Mar	LNO	4.4/F	–	–	–
IND-30-2010	Mar	LNO	6.0/M	–	–	–
IND-34-2010	Mar	KRI	8.0/M	–	+	–
IND-35-2010	Mar	STP	1.5/M	+	+	–
IND-36-2010	Mar	STP	4.0/M	+	–	–
IND-43-2010	May	LNO	2.5/M	+	+	–
IND-37-2010	May	STP	2.3/F	–	+	–
IND-38-2010	May	STP	2.8/M	+	+	–
IND-42-2010	May	STP	4.7/M	–	–	+
IND-45-2010	May	STP	9.0/M	–	+	–
IND-20-2010	Aug	KRI	4.0/M	+	+	–
IND-21-2010	Aug	KRI	8.0/M	–	–	–
IND-12-2010	Apr	KRI	0.9/F	+	+	–
IND-17-2010	Apr	KRI	2.0/M	–	+	–
B						
IND-23-2010	Jul	HDO	2.0/F	+	+	+
IND-24-2010	Jul	HDO	2.5/M	+	+	–
C						
IND-14-2010	Jul	KRI	1.4/M	+	+	–
IND-16-2010	Jul	KRI	2.0/M	+	+	–
IND-19-2010	Jul	KRI	2.5/F	+	+	–
IND-18-2010	Jul	KRI	3.0/F	–	+	–
D						
IND-15-2010	Jul	KRI	6.5/M	+t	+	+
IND-22-2010	Aug	HDO	4.5/M	–	–	–
IND-40-2010	Aug	RBL	2.0/M	–	–	–
IND-39-2010	Aug	LNO	4.0/F	–	+	–
IND-41-2010	Aug	LNO	4.5/M	+	–	+
IND-44-2010	Aug	LNO	3.0/M	+	+	–
IND-33-2010	Aug	HDO	2.0/F	+	+	+
IND-32-2010	Aug	HDO	2.0/M	–	+	–

*AFP, acute flacid paralysis; LNO, Lucknow; STP, Sitapur; KRI, Lakhimpur-Kheri; HDO, Hardoi; RBL, Raebareli; –,absence of clinical feature; + presence of clinical feature. †Different clades (Figure) of EV13 identified in the study. ‡Designation of investigated isolates of all Uttar Pradesh 2010 EV13 isolates. §Month of isolation. ¶District of AFP patient.

**Table 2:** Clinical characteristics of 45 AFP case-patients with nonpolio enterovirus serotypes, Uttar Pradesh, India, 2010*

Sign/symptom	AFP case-patients with serotype, no. (%)							
	E13, n = 30	E25, n = 4	E7, n = 2	E4, n = 1	E33, n = 1	E75, n = 2	CB6, n = 2	CB3, n = 3
Fever at onset of paralysis	15 (50)	3 (75)	–	–	1 (100)	2 (100)	–	3 (100)
Asymmetric paralysis	20 (67)	2 (50)	1 (50)	1 (100)	–	2 (100)	2 (100)	2 (67)
Residual paralysis	7 (23)	–	–	1 (100)	–	1 (50)	–	–

*AFP, acute flacid paralysis, E, echovirus;EV, enterovirus; CB, coxsackievirus B; –, absence of clinical feature.

The 244-nt partial VP1 gene sequence of all the Uttar Pradesh E13 isolates shared 75.4%–79.9% nt identity and 87.7%–93.8% aa identity with the prototype E13 strain, Del Carmen (GenBank accession no. AY302539). Phylogenetic analysis indicated the existence of 2 different Uttar Pradesh EV13 clades in the neighbor-joining tree (Figure). Uttar Pradesh 2010 EV13 viruses in the largest clade segregated into 3 groups (labeled A–C, Figure), whereas the second large 2010 Uttar Pradesh EV13 clade was monophyletic (labeled D, Figure). Partial VP1 sequences of Uttar Pradesh EV13 clade A shared 96.5%–100% nt identity (100% aa identity) with one another. Clade B shared 89.4%–90.7% nt identity (95.5%–98.8% aa identity) with clade A and 84.2%–84.5% nt identity (97.5% aa identity) with clade C. Clade C shared 88.9%–88.1% nt identity (95.5%–98.8% aa identity) with clade A. Uttar Pradesh EV13 clade D shared 73%–76% nt identity (90.1%–93.5% aa identity) with clades A, B, and C. Although variation exists among the partial VP1 sequences of EV13 strains from India, the EV13 clades A, B, and C appear to be closely related to earlier India isolates (2007 and 2008) followed by the isolates from Pakistan (2009), Bangladesh (2000), and the Republic of Georgia (2004). The EV13 D clade was genetically more distant and grouped together with year 2000 strains from India and Bangladesh (isolated in 1999), and 1 isolate from Georgia (isolated in 2004).

**Figure:**
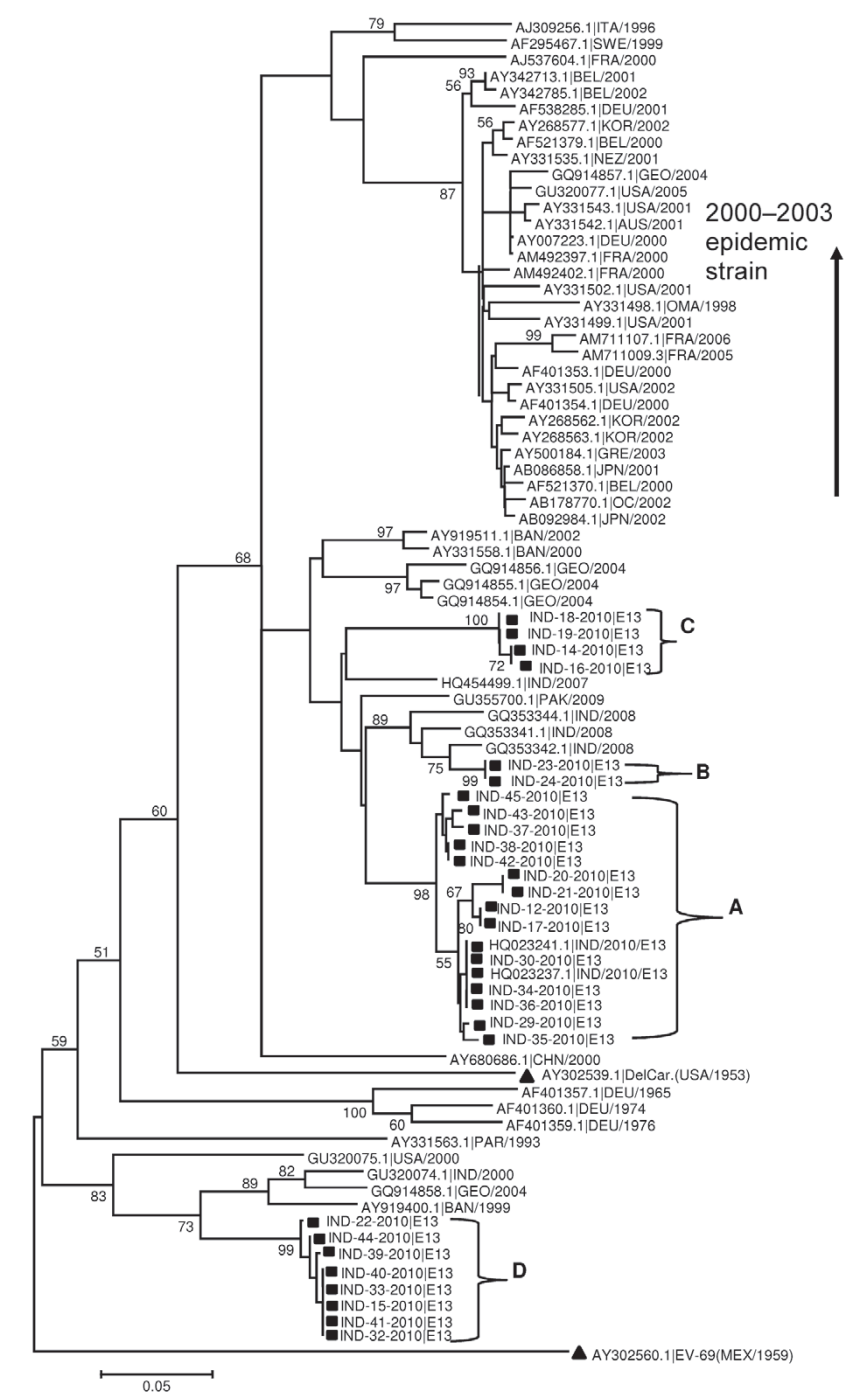
Phylogenetic tree based on alignments of partial viral protein 1 gene sequences of echovirus 13 (E13) constructed by the neighbor-joining method implemented in MEGA version 5.05 software (*7*) by using the Kimura-2 parameter nucleotide substitution model. Bootstrap analysis included 1,000 pseudoreplicate datasets. Clusters are labeled A, B, C, and D. Square indicates Uttar Pradesh E13 from fecal samples. All Uttar Pradesh E13 isolates on the tree are identified by using the same numbers listed in Table 1. Triangle indicates E13 prototype Del Carmen and the out-group enterovirus 69. Scale bar indicates nucleotide substitutions per site.

## Conclusions

We demonstrated the utility of a modified WHO laboratory protocol by combining classical and molecular methods for rapid detection and identification of nonpolio HEV (2,*4*–*6*). Identifying the EV in mixed EV infections is challenging when this protocol is used, but on balance, pan-EV testing followed by VP1 RT-PCR and sequencing delivers rapid results and more efficient use of labor and resources than does the traditional method and enables identification of newer EV types.

Numerous reports have described AFP patients infected with EVs, coxsackieviruses, and newer numbered EVs (*3*,*8*). Nonpolio HEVs also have been detected in epidemics of paralytic disease (*9*). Although nonpolio HEVs can be isolated from asymptomatic children, few studies have compared the nonpolio HEV serotypes found in AFP case-patients and in healthy children. Identifying nonpolio HEV serotypes associated with AFP in India provides useful EV surveillance data and should be explored further in conjunction with virologic evaluation of appropriate community controls and additional clinical specimens from AFP case-patients (cerebrospinal fluid, throat swab samples, serum), which could indicate the nonpolio HEV type causing paralytic disease.

We identified 8 nonpolio HEV serotypes (E4, E7, E13, E25, E33, EV75, CVB3, and CVB6). E13, the predominant serotype, has been isolated rarely (*3*,*10*); however, nonpolio HEVs have been reported from India in connection with sporadic and epidemic meningitis/encephalitis cases (*11*,*12*). A wide spectrum of illnesses have been reported with E13 (*13*), so finding E13-associated AFP cases mimicking poliomyelitis (*14*) is not surprising.

The global aseptic meningitis epidemic caused by EV13 during 2000–2003 was associated with a single E13 genotype (*15*); however, our findings indicate that several different and genetically diverse E13s have been cocirculating in India. The Uttar Pradesh E13 partial VP1 gene sequences are most closely related to recent E13 strains from central and southern Asia. The high genetic diversity among the Uttar Pradesh E13 isolated from patients in a local area over 6 months implies a continuous pattern of circulation. High-density human populations, immunologically susceptible cohorts, and nonhygienic environmental conditions probably facilitated the E13 genetic heterogeneity in our study.

Although we report a high prevalence of E13 infection in AFP case-patients, our study is restricted by the modest number of cases from a small geographic area sampled over a short period. The epidemiologic and nonpolio HEV genetic information gathered warrants further studies in larger groups of children to gain better insight into AFP caused by nonpolio HEV and to identify the etiologic nonpolio HEV type(s) associated with sporadic or epidemic AFP as India nears the goal of polio elimination.
